# Expression profile of long noncoding RNAs and comprehensive analysis of lncRNA-*cis*TF-DGE regulation in condyloma acuminatum

**DOI:** 10.1186/s12920-024-01938-z

**Published:** 2024-06-20

**Authors:** Bo Xie, Yinhua Wu, Su Wang, Liming Ruan, Xiaoyan Liu

**Affiliations:** 1 Department of Urology, the First Affiliated Hospital, Zhejiang University School of Medicine, 79# Qingchun Road, Hangzhou, Zhejiang Province 310003 China; 2 Department of Dermatology, the First Affiliated Hospital, Zhejiang University School of Medicine, 79# Qingchun Road, Hangzhou, Zhejiang Province 310003 China; 3 Department of Dermatology, Beilun People’s Hospital of Ningbo City, 1288# Lushan East Road, Ningbo, Zhejiang Province 310058 China

**Keywords:** Human papillomavirus, Condyloma acuminatum, Long noncoding RNA, Bioinformatics

## Abstract

**Objective:**

To identify differentially expressed long noncoding RNAs (lncRNAs) in condyloma acuminatum (CA) and to explore their probable regulatory mechanisms by establishing coexpression networks.

**Methods:**

High-throughput RNA sequencing was performed to assess genome-wide lncRNA expression in CA and paired adjacent mucosal tissue. The expression of candidate lncRNAs and their target genes in larger CA specimens was validated using real-time quantitative reverse transcriptase polymerase chain reaction (RT‒qPCR). Furthermore, Gene Ontology (GO) and Kyoto Encyclopedia of Genes and Genomes (KEGG) analyses were used for the functional enrichment analysis of these candidate lncRNAs and differential mRNAs. The coexpressed mRNAs of the candidate lncRNAs, calculated by Pearson’s correlation coefficient, were also analysed using GO and KEGG analysis. In addition, the interactions among differentially expressed lncRNAs (DElncRNAs)-*cis*-regulatory transcription factors (cisTFs)-differentially expressed genes (DEGs) were analysed and their network was constructed.

**Results:**

A total of 546 lncRNAs and 2553 mRNAs were found to be differentially expressed in CA compared to the paired control. Functional enrichment analysis revealed that the DEGs coexpressed with DElncRNAs were enriched in the terms of cell adhesion and keratinocyte differentiation, and the pathways of ECM-receptor interaction, local adhesion, PI3K/AKT and TGF-ß signaling. We further constructed the network among DElncRNAs-*cis*TFs-DEGs and found that these 95 DEGs were mainly enriched in GO terms of epithelial development, regulation of transcription or gene expression. Furthermore, the expression of 3 pairs of DElncRNAs and *cis*TFs, EVX1-AS and HOXA13, HOXA11-AS and EVX1, and DLX6-AS and DLX5, was validated with a larger number of specimens using RT‒qPCR.

**Conclusion:**

CA has a specific lncRNA profile, and the differentially expressed lncRNAs play regulatory roles in mRNA expression through *cis*-acting TFs, which provides insight into their regulatory networks. It will be useful to understand the pathogenesis of CA to provide new directions for the prevention, clinical treatment and efficacy evaluation of CA.

**Supplementary Information:**

The online version contains supplementary material available at 10.1186/s12920-024-01938-z.

## Introduction

Condyloma acuminatum (CA), mostly caused by low-risk HPV6 or HPV11, is characterized by repeated recurrence [1] and rapid proliferation [2]. The mechanisms by which HPV promotes cell proliferation and inhibits apoptosis of host cells include the following: on the one hand, HPV E6/E7 protein reduces apoptosis and autophagy by inhibiting p53/Rb; on the other hand, it activates the PI3K/Akt/mTOR and p38 MAPK/ERK pathways to promote cellular proliferation [3]. By recognizing EGFR, the HPV E5 protein activates the PI3K/Akt/MEK/Erk1/2 pathway [4] and enhances the transgenic activity of E7 [5]. E6/E7 of low-risk HPV also exerts similar activity to that of high-risk E6/E7 and affects common downstream pathways [6]. However, the upstream regulatory mechanisms of these pathogenic pathways for CA remain unknown.

Long noncoding RNAs (lncRNAs), more than 200 nucleotides, are described as RNAs with little or no protein-coding capability. These lncRNAs are involved in almost all important regulatory processes, including chromosome silencing or remodelling, transcriptional or posttranscriptional processing and intranuclear transportation [7, 8]. However, the significance of the expression and function of lncRNAs and their probable mechanism in CA has not been illuminated.

We speculated that lncRNAs expressed differentially in CA might regulate downstream target genes and participate in the occurrence and development of CA. To verify this hypothesis, high-throughput RNA sequencing was used to detect the genome-wide expression levels of lncRNAs and mRNAs between CA and their paired adjacent normal mucosa. We further validated the candidate lncRNAs in a larger number of CA specimens using real-time quantitative reverse transcriptase polymerase chain reaction (qRT‒PCR). Moreover, bioinformatics for differentially expressed genes (DEGs) using Gene Ontology (GO) terms and Kyoto Encyclopedia of Genes and Genomes (KEGG) annotation were performed to explore the probable regulatory mechanisms of candidate lncRNAs. Furthermore, we analysed the interactions among differentially expressed lncRNAs (DElncRNAs)-cis-regulatory transcription factors (cisTFs)-DEGs, focusing on *cis*-regulatory mechanisms. Studying lncRNAs in CA may be helpful to provide a novel understanding of the pathogenesis of CA and to explore potential intervention targets for CA.

## Materials and methods

### Patients and specimens

Approval for this study was obtained from the Institutional Review Board of the First Affiliated Hospital, School of Medicine of Zhejiang University (2018-086 and 2022 -795). Written informed consent was obtained from all subjects. Female CA patients aged 20–40 years old with positive HPV6 or HPV11 genotyping were included. A total of 5 pairs of HPV-positive CA specimens (CA group) and their adjacent mucosal tissue (CON group) were used for high-throughput transcriptional sequencing. Another 40 CA specimens (CA group) and 15 HPV-negative vaginal mucosal tissues (CON group) were collected for further verification using qRT‒PCR. An HPV GenoArray Test Kit (Hybribo, China) was used to detect HPV genotyping. CA patients with tumors and other immunocompromised diseases were excluded.

### RNA extraction and transcriptome sequencing

All samples were stored in liquid nitrogen immediately after sampling. Then, the TRIzol reagent (Invitrogen, CA, USA) was used for extraction of total RNA. The RNA amount and purity of each sample was quantified using NanoDrop ND-1000 (NanoDrop, Wilmington, DE, USA) and the RNA integrity was assessed by Bioanalyzer 2100 (Agilent, CA, USA) with RIN number > 7.0. The Epicenter RiboZero Gold Kit (Illumina, San Diego, USA) was used for deletion of ribosomal RNA. Then, the final cDNA library was established by reverse transcription of the poly(A)- or poly(A) + RNA following the protocol for the mRNA-seq specimen preparation kit (Illumina, San Diego, USA). The average insert size for the final cDNA library was 300 ± 50 bp. Then, the 2 × 150 bp paired-end sequencing (PE150) was performed using the Illumina HiSeq 2000 platform at LC-Bio Biotech, Ltd. (Hangzhou, China). The sequencing data were submitted to the GEO database with the accession number GSE172140.

### Read alignment and DEG analysis

Several strategies were applied to obtain clean reads as follows: The adapter of mRNA was removed and reads > 17 bp were retained; The first three bases were cut off; The bases < Q20 were removed and reads > 16 bp were retained; The bases containing 30% bases < Q20 were removed; NN ( unsure bases in sequencing ), poly G and poly A were discarded. Clean reads were aligned to the human GRch38 genome by HISAT2 [9], allowing 4 mismatches. Based on the uniquely mapped reads, the StringTie (https://ccb.jhu.edu/software/stringtie) was used to perform expression level for all mRNAs from input libraries by calculating the fragments per kilobase of transcript per million fragments mapped (FPKM) for each gene. DEGs were identified using edgeR software [10] and screened with a cut-off of fold change (FC) $\ge$ 2 or $\le$ 0.5 and false discovery rate (FDR) < 0.05 as the thresholds to determine whether genes were differentially expressed.

### LncRNA prediction

**Fig. 1:**
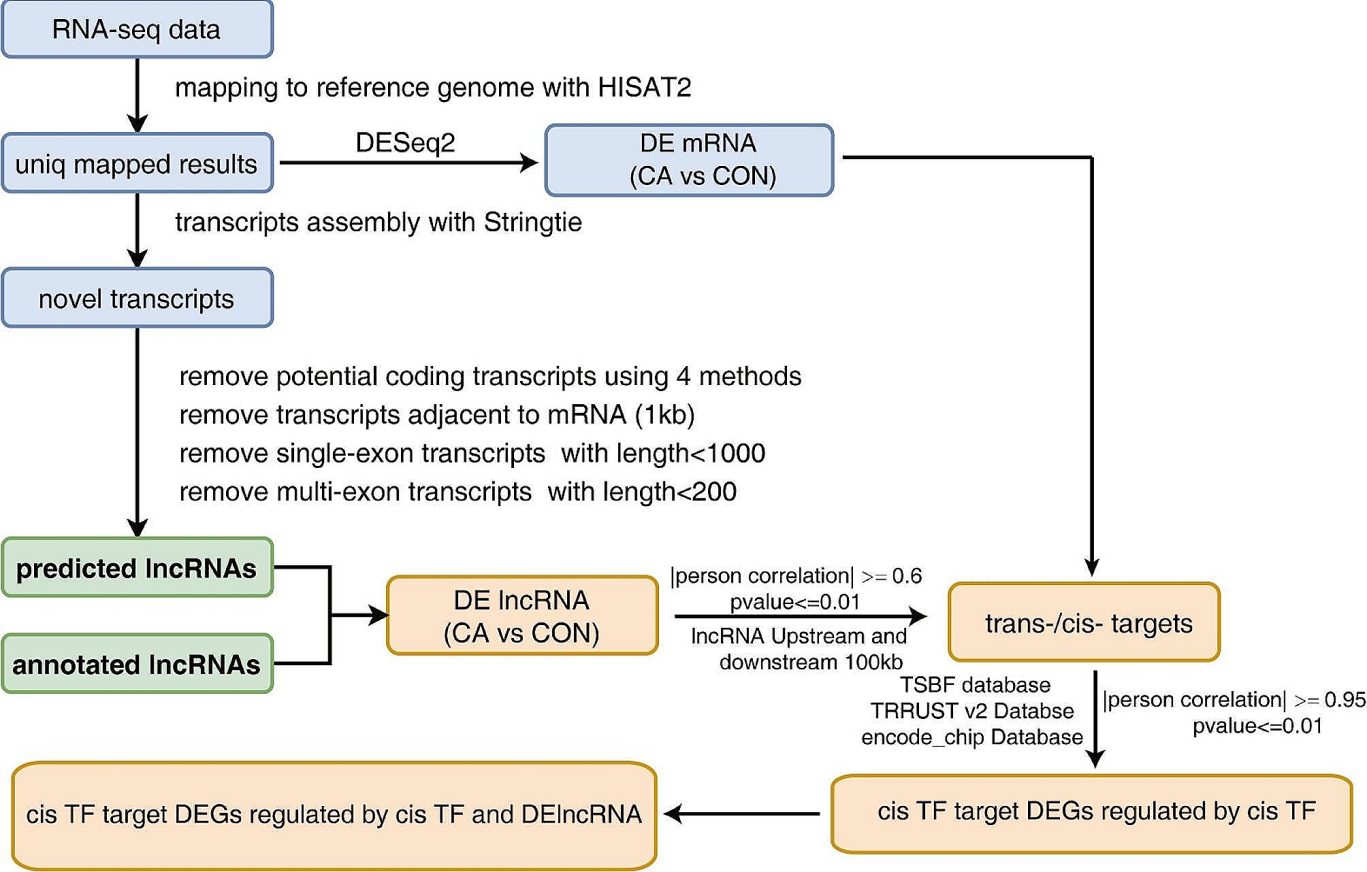
Workflow of lncRNA prediction and analysis in this research

Four software programs of Coding Potential Calculators (CPC2) [11], ORF Length and GC Content (LGC) [12], Coding-Non-Coding Index (CNCI) [13] and Coding Potential Assessment Tool (CPAT) [14], were used to predict or identify credible lncRNAs and for subsequent analysis and processing. In brief, the workflow of lncRNA prediction and further analysis of lncRNA *trans*-/*cis*-targets is shown in Fig. 1. Meanwhile, four types of novel transcripts were also removed, as shown in Fig. 1.

### Functional enrichment analysis

Functional enrichment of GO terms and KEGG pathways for all DEGs was performed using the KOBAS 2.0 server [15]. The hypergeometric test and Benjamini‒Hochberg FDR controlling procedure were used to define the enrichment of each term. Reactome pathway analysis was also performed online using the Reactome database (http://reactome.org).

### **LncRNA*cis*-regulatory target**

The threshold of colocation was set as 100 kb upstream and downstream of lncRNAs in the *cis*-regulatory relationship pair [16]. For screening the lncRNA target pairs, the Pearson correlation coefficient between lncRNA and mRNA colocalization was calculated, and the absolute value of the correlation coefficient was set as greater than 0.6 and *P* value < 0.01. Then, the intersection of the two datasets of colocalization and coexpression was used to obtain the *cis* target of lncRNA. Then, lncRNA *cis*-regulatory transcription factors (*cis*TFs) were filtered out according to a catalog of 1796 transcription factors (TFs) retrieved from HumanTFDB (http://bioinfo.life.hust.edu.cn/HumanTFDB#!/).

### Coexpression analysis between TFs and DEGs

Next, we also calculated the Pearson correlation coefficient between *cis*TFs and DEGs, and the absolute value of the correlation coefficient between *cis*TF-DEG relationship pairs was also set as correlation coefficient > 0.95 and *P* value < 0.01. Annotated *cis*TF-target pairs were downloaded from three databases: Transcription Factor Target Gene Database (http://tfbsdb.systemsbiology.net/download), TRRUST v2 Database (https://www.grnpedia.org/trrust/) and encode_chip TF targets Database. Furthermore, we generated a regulatory network between lncRNAs, *cis*TF, and DEmRNAs using Cytoscape software [17]. In brief, the excel of DElncRNA_TF_Target was imported into the Cytoscape, and the Source node, Target node, Source Node Attribute and Target Node Attribute according to the four column name were selected to layout the network of DElncRNA-*cis*TF-DEGs.

### RT‒PCR for quantification of candidate lncRNAs

Primers for candidate lncRNAs and GAPDH were synthesized by RiboBio (Guangzhou, China). A 10 µL reaction volume containing 5 µL of 2 × SYBR® Premix Ex Taq™ and 0.2 µL of 50 × ROX reference dye (TaKaRa) was used for the amplification reactions. The ∆Ct data were collected automatically, and the data of -∆∆Ct were calculated by -∆∆Ct = average ∆Ct of the negative control group-∆Ct of the treated group. The relative expression of a target gene was calculated using 2-^∆∆Ct^. Each treatment was performed in triplicate.

### Statistical analysis

The R package factoextra was used for PCA, and the samples were clustered using the first two principal components. Next-generation sequencing data and genome annotations were visualized using in-house scripts (sogen) after normalization of the reads by RPM (Reads per million mapped reads) for each gene: RPM = ExonMappedReads × 10^6^ ÷ TotalMappedReads. Hierarchical clustering based on Euclidean distance was performed using the pheatmap package. Student’s t test was used to compare the expression of target genes between CA group and CON group. Other statistical results were obtained by R software, where a *P* value < 0.05 was considered statistically significant.

## Results

### Differential expression profiles of lncRNAs and mRNAs in CA specimens

**Fig. 2:**
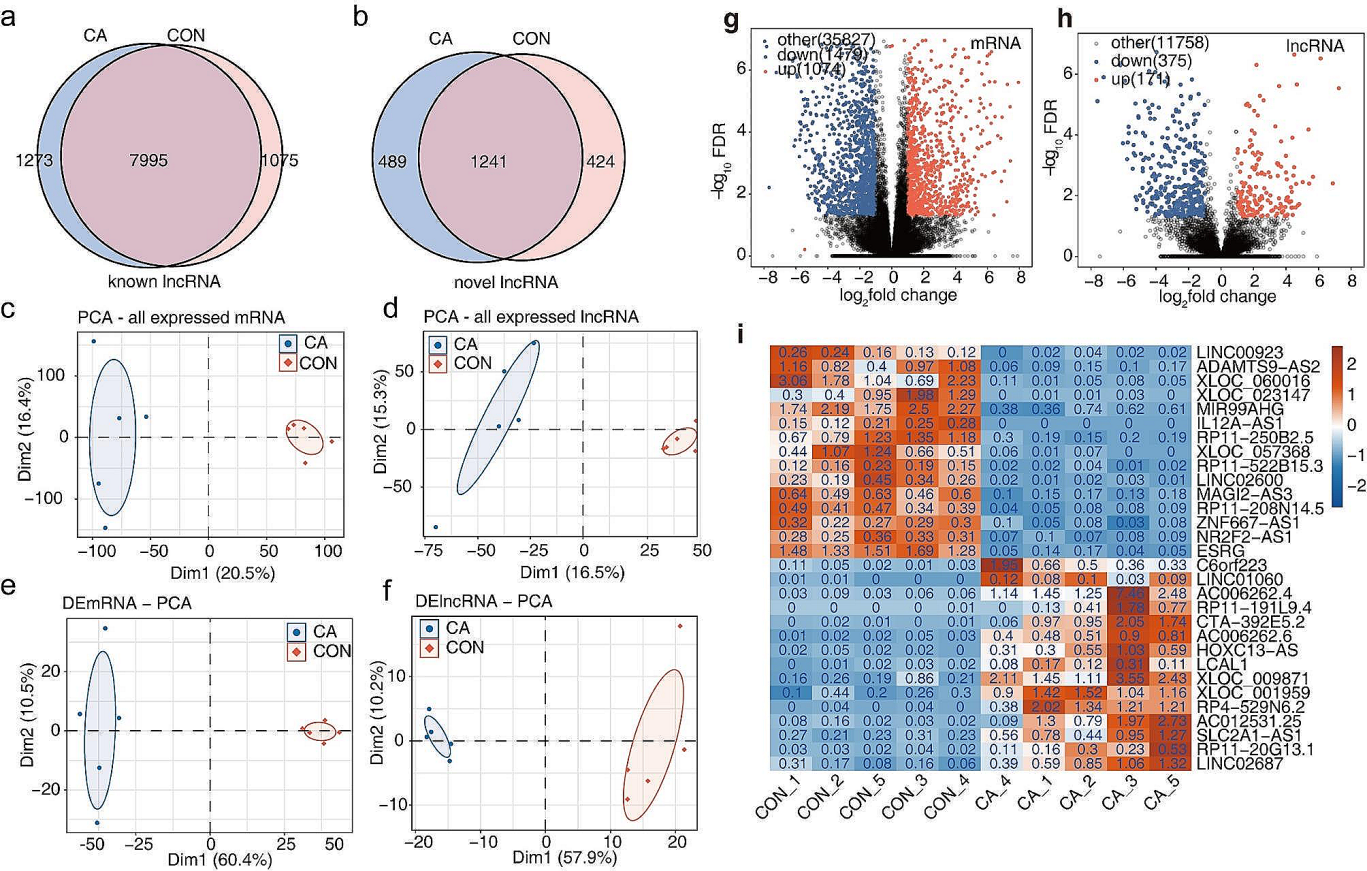
Identification of differentially expressed lncRNAs in condyloma acuminatum. (**a-b**) Venn diagram showing the number of detected lncRNAs. The known and new predicted lncRNAs were detected (expressed in at least 1 sample with FPKM > 0). (**c-f**) PCA based on the FPKM value of all mRNAs, all lncRNAs, differentially expressed mRNAs and differentially expressed lncRNAs. The ellipse for each group is the confidence ellipse. (**g-h**) Volcano plot showing differentially expressed mRNAs and lncRNAs between the CA group and the CON group, with cut-offs of DEseq2. FDR < 0.05 and FC ≥ 2 or ≤ 0.5. (**i**) Heatmap showing the expression profile of the top 15 significantly upregulated and downregulated lncRNAs

As shown in Fig. 2a, b and a total of 10,343 known lncRNAs and 2154 novel predicted lncRNAs were detected (expressed in at least 1 sample with FPKM > 0). The Venn diagram in Fig. S1a shows the overlapping lncRNAs by the four methods of CPC2, LGC, CNCI and CPAT, and the pie chart in Figure S1b based on the lncRNA type distribution shows that 3609 were intronic lncRNAs (84%), 471 were antisense lncRNAs (11%), and 216 were intergenic lncRNAs (5%). The distribution of exon length and density of the length distribution of noncoding RNAs and protein-coding RNAs are displayed in Fig. S1c-d.

Principal component analysis (PCA) for the expression pattern in these ten samples showed that all expressed genes and DEGs in CA and CON samples were clearly separated (Fig. 2c and f), indicating that the gene expression profile was different between CA and CON samples. Next, the differences in lncRNAs and mRNAs with a cut-off of FC ≥ 2 or ≤ 0.5 between the CA and CON groups were displayed using volcano plot filtering (Fig. 2g and h). A total of 2553 mRNAs were differentially expressed, with 1074 upregulated and 1479 downregulated (Fig. 2g). Meanwhile, a total of 546 lncRNAs, 171 upregulated and 375 downregulated, were found to be differentially expressed between CA and CON (Fig. 2h). The top 15 significantly upregulated and downregulated lncRNAs are displayed using a heatmap in Fig. 2i.

### Functional analysis of DEGs and DEGs coexpressed with DElncRNAs using GO or KEGG annotations

**Fig. 3:**
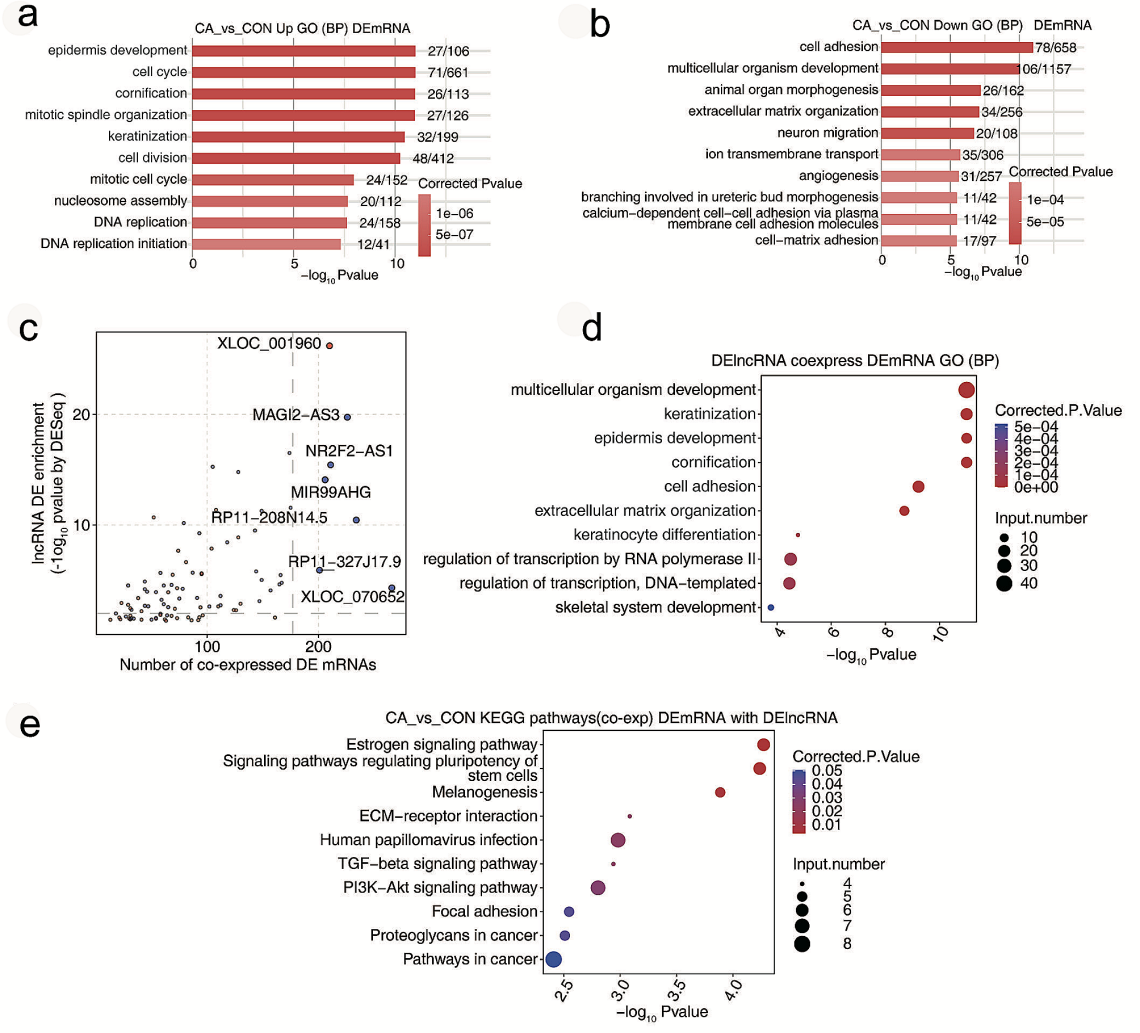
Analysis of differential expression of lncRNAs and mRNAs in condyloma acuminatum. (**a-b**) Bar plot showing the most enriched GO terms (biological process) for the upregulated and downregulated DEGs. (**c**) Scatter plot showing DElncRNA enrichment by CA compared with the CON samples and the number of coexpressed DEmRNAs. Red points represent upregulated lncRNAs involved in coexpression pairs, and blue points represent downregulated lncRNAs. Cut-offs of *P* value < 0.01 and Pearson coefficient > 0.9 were applied to identify the coexpression pairs. (**d-e**) Bubble diagram exhibiting the most enriched GO terms (biological process) and KEGG pathways of the DEmRNAs coexpressed with DElncRNAs

The functions of most lncRNAs have not been investigated completely until now, and analysing differential mRNAs with GO functional analysis and KEGG pathway analysis might be helpful to recognize the role of lncRNAs. In the present study, GO terms of BP for upregulated DEGs in CA were involved in the cell cycle, cell division and mitotic cell cycle (Fig. 3a), and those for downregulated DEGs in CA were involved in cell adhesion and angiogenesis (Fig. 3b). Meanwhile, the top 10 pathways for upregulated and downregulated DEGs using KEGG analysis are displayed in Fig. S2c-2d, including cell cycle and ECM-receptor interaction, PI3K/AKT signaling and TGF-ß signaling pathways.

Furthermore, the Pearson correlation coefficient between DEGs and DElncRNAs was calculated based on the transcriptional sequencing data, and the DEGs coexpressed with the candidate lncRNAs with correlation coefficients > 0.90 (*P* < 0.01) were annotated by GO terms or KEGG. Figure 3c displays the DElncRNA enrichment by CA compared with the CON samples and the number of coexpressed DEmRNAs. Interestingly, GO and KEGG annotations showed that DEGs coexpressed with DElncRNAs were also enriched in cell adhesion, keratinocyte differentiation (Fig. 3d), and the pathways involved in ECM-receptor interaction, PI3K/AKT signaling, TGF-ß signaling and local adhesion (Fig. 3e). This result indicated that these DElncRNAs in CA tissues might play potential regulatory roles in these pathways.

### Construction of the regulatory network among DElncRNAs-*cis*TF-DEGs in CA

**Fig. 4:**
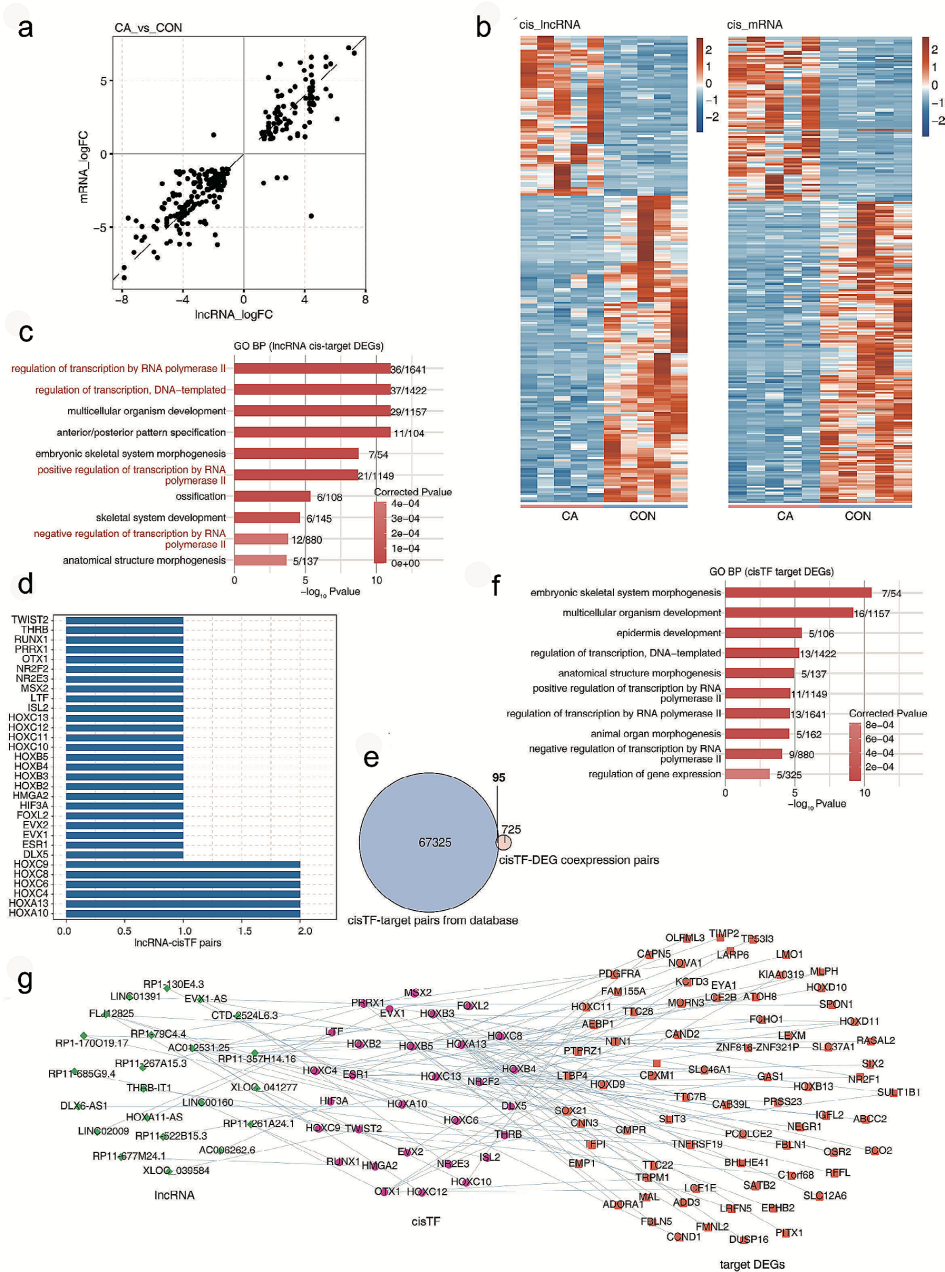
*Cis*-regulatory TFs of DElncRNAs associated with condyloma acuminatum. (**a**) Scatter plot showing the log2 FC of DElncRNAs in CA compared with CON samples and their *cis*-regulatory genes. (**b**) Heatmap showing lncRNAs and *cis*-regulatory genes. (**c**) Bar plot showing the most enriched GO terms (biological process) of the differential lncRNA *cis*-target DEGs. (**d**) Bar plot showing the number of lncRNA-*cis*TF pairs. (**e**) Venn diagram showing the number of overlapping pairs of *cis*TF-target pairs from the database and *cis*TF-DEG coexpression pairs in the present study. (**f**) Bar plot showing the most enriched GO terms (biological process) of the *cis*TF target DEGs. (**g**) Network diagram showing the *cis*TF-targeted DEGs regulated by *cis*TF and DElncRNAs

LncRNAs play critical roles in gene regulation through mechanisms in *cis* or in *trans* [18]. One of our objectives in the present study was to identify potential *cis*-regulation of DElncRNAs. Figure 4a shows a positive relationship between DElncRNAs and *cis*-regulatory DEGs, whose levels are displayed using a heatmap in Fig. 4b. Next, we analysed the GO term enrichment and KEGG of these *cis*DEGs by lncRNAs and found that they were mainly enriched in transcription-related biological functions (Fig. 4c) and that most *cis*TFs belonged to the HOX family (Fig. 4d). Thus, we speculated that a large number of TFs, especially those in the HOX family, may be regulated by lncRNAs in CA. Through the intersection of these *cis*TF potential target genes from the database and *cis*TF coexpressed DEGs in our data, 95 DEGs were identified as possible targets of these *cis*TFs (Fig. 4e). The GO term annotation analysis for these 95 DEGs revealed that these genes were mainly enriched in GO terms of epithelial development, regulation of transcription and regulation of gene expression (Fig. 4f). In addition, a regulatory network among DElncRNAs-*cis*TF-DEGs was constructed, as shown in Fig. 4g.

**Fig. 5:**
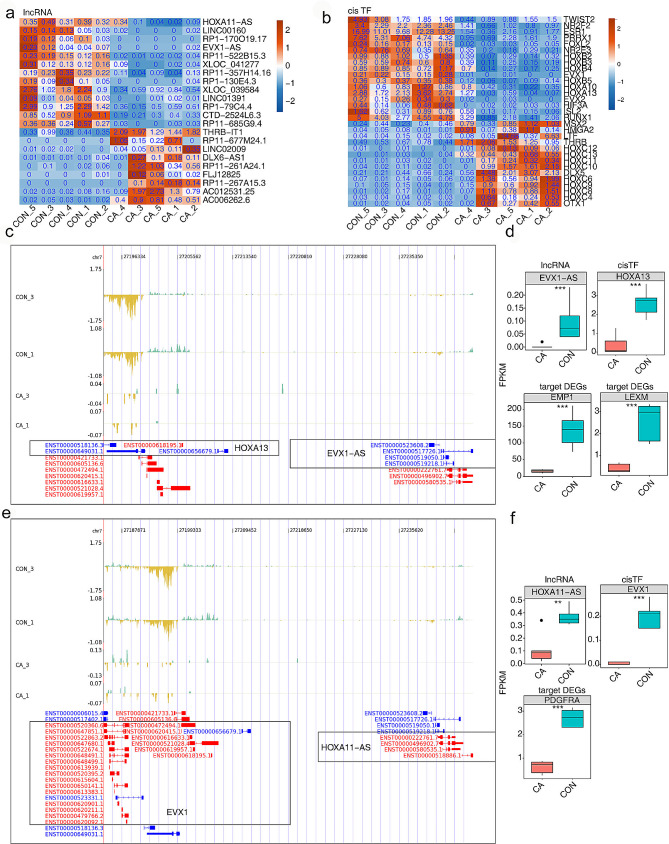
Expression of DElncRNAs, *cis*TFs and targeted DEGs in CA and CON samples. (**a-b**) The heatmap showing the expression profile of the DElncRNAs and cisTFs in Fig. 4g. (**c-d**) The read distribution showed lncRNA EVX1-AS and its *cis*-regulated TF HOXA13. The upward and green reads in the upper part represent for the red transcripts in the bottom part, and the downward and yellow reads represent for the blue transcripts in the bottom part. Boxplot showing the expression of lncRNAs, *cis*TFs and target DEGs. *** *P* < 0.001. (**e-f**) Read distribution showing lncRNA HOXA11-AS and its *cis*-regulated TF EVX1. The upward and green reads in the upper part represent for the red transcripts in the bottom part, and the downward and yellow reads represent for the blue transcripts in the bottom part. Boxplot showing the expression of lncRNAs, their regulated *cis*TF and target DEGs. **P < 0.01, *** *P* < 0.001

**Fig. 6:**
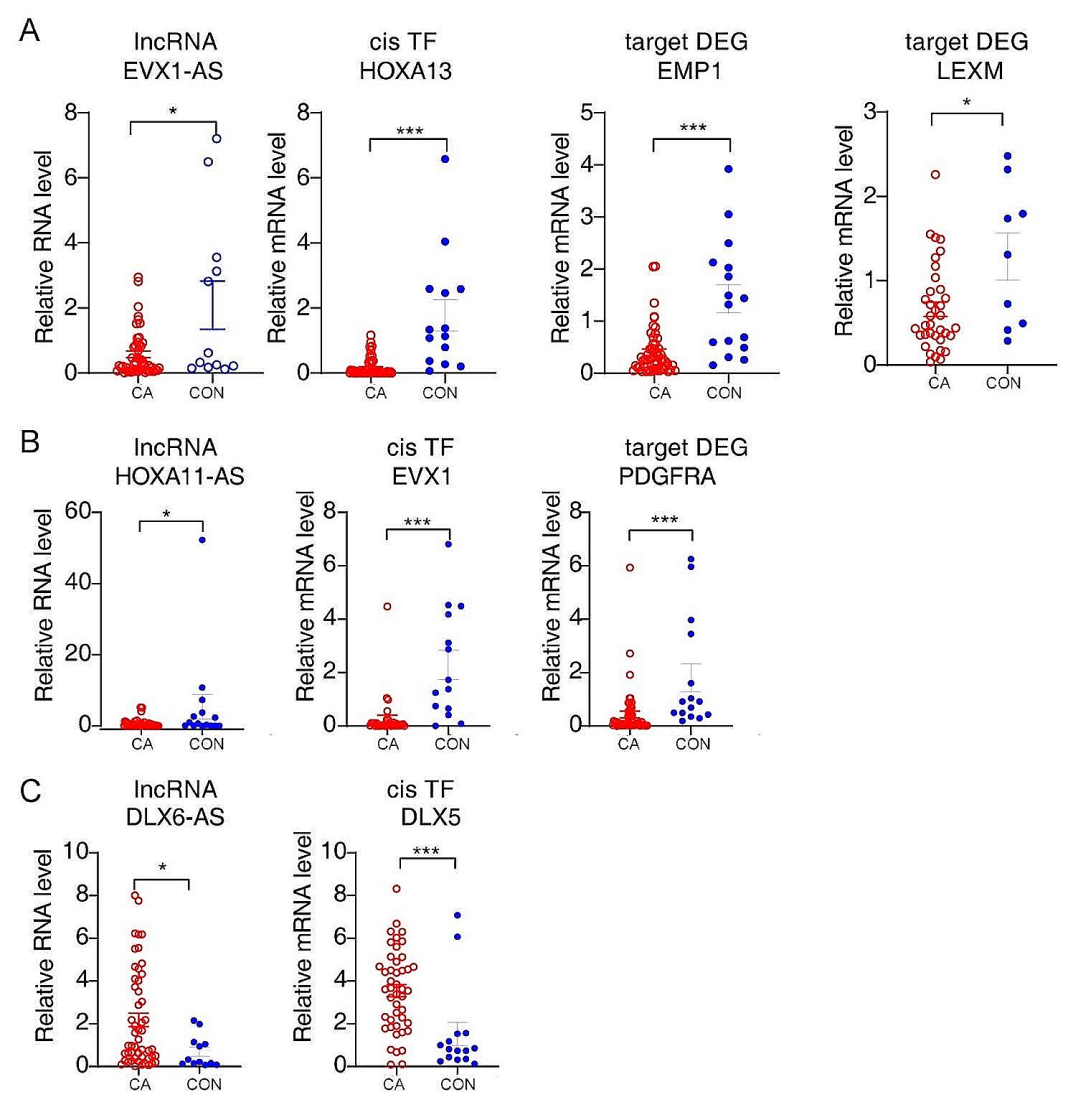
Validation of target lncRNAs and the*ir cis*-regulatory TFs and DEGs in condyloma acuminata. (**a-c**) Bar plots showing the relative expression levels of the lncRNAs, *cis*TFs and TF-target DEGs using qRT‒PCR. Error bars represent the mean ± SEM. * *P* < 0.05, ** *P* < 0.01, *** *P* < 0.001

In Fig. 5a-b, the expression of lncRNAs and *cis*TFs from the DElncRNA-*cis*TF-DEG network, as shown in Fig. 4g, is displayed using a heatmap. Based on the number of lncRNA-*cis*TF-DEG pairs and the probable functions of target lncRNAs or their *cis*TFs, we selected several lncRNAs of EVX1-AS, HOXA11-AS and DLX6-AS and showed the read distribution for these target lncRNAs and their *cis*TFs (Fig. 5c and e and S3a). Furthermore, based on the FPKM value, the expression of EVX1-AS, its *cis*TF HOXA13, and its target DEGs EMP1 and LEXM all decreased in the CA group (Fig. 5d). The expression of HOXA11-AS, EVX1 and PDGFRA was also attenuated in the CA group (Fig. 5f), while that of DLX6-AS and DXL5 was increased in the CA group (Fig. S3b). Then, the expression levels of these target lncRNAs and *cis*-regulatory DEGs were validated in 40 cases of CA compared with 15 cases of normal vaginal mucosal tissues using real-time qRT‒PCR. The qRT‒PCR results revealed a significant decrease in EVX1-AS, HOXA13, EMP1, LEXM, HOXA11-AS, EVX1 and PDGFRA and an obvious increase in DLX6-AS and DXL5 in CA tissues compared to CON tissues (Fig. 6a-c), which was in agreement with the RNA-seq analysis. Both our sequencing data and the qRT‒PCR verification results indicated the probable *cis*-regulatory mechanisms of lncRNAs-*cis*TFs-DEGs in CA pathogenesis.

## Discussion

LncRNAs are a novel class of regulatory noncoding RNAs that participate in almost all biological or pathological processes [18]. Studies on HPV-related lncRNAs have mainly focused on the pathogenesis, invasion and metastasis of cervical cancers (CCs) and head and neck squamous cell carcinoma (HNSCC) caused by high-risk HPV. As early as 2016, Chinese scholars reported that in HPV18 + SiHa or HPV16 + HeLa cell lines, certain lncRNAs led to differential expression of their target mRNAs involved in several crucial processes of DNA repair, cellular cycle, proliferation and apoptosis [19]. In 2017, another Chinese group revealed the noncoding RNA profiles in HPV16-positive CC, compared them to adjacent tissue and constructed a coexpression and ceRNA network among differential lncRNAs. circRNAs, miRNAs and mRNAs [20]. In HNSCC, 132 different lncRNAs were identified under different HPV-infected states of HNSCC, among which *HOTAIR*, *PROM1*, *CCAT1* and *MUC19* were negatively correlated to myeloid-derived suppressor cell recruitment in HPV-associated HNSCC [21]. In recent years, the functions of several specific HPV-related lncRNAs have been identified, most of which are mediated by oncogenic HPV E6/E7 and promote cervical cancer progression [22–24]. Recently, the increase in lnc-*FANCI-2* mediated primarily by E7 was shown to be due to YY1’s interaction with an E7 CR3 core motif and activation of the promoter of *lnc-FANCI-2* [25]. In addition, HPV18 delayed replicative senescence of human keratinocytes through downregulation of antisense noncoding mitochondrial RNA-2 [26].

There have been very few studies on the transcriptome in CA specimens mainly caused by low-risk HPV. Dr. Zhang and coworkers compared the gene expression between the stable HPV-6bE7 HaCaT cell line and parental cells [27], and Gao et al. reported proteomic changes in CA tissue caused by mild hyperthermia treatment [28]. However, neither of them detected lncRNAs that were differentially expressed in CA. Although Tarkhan AH and coworkers reported lncRNA profiles in LR-HPV infection in 2022, their specimens were limited to common warts without a larger cohort for validation [29]. In the present study, we not only revealed transcriptional profiles, including lncRNAs and mRNAs but also analysed the probable regulatory pathway and *cis*-regulatory mechanism by lncRNAs in CA for the first time, further confirming the sequencing data in larger samples using qRT‒PCR. Herein, we used CPC2, LGC, CNCI [13] and CPAT [14] to predict or identify credible lncRNAs. CPC2 is a tool to evaluate the protein coding potential of transcript by comparing the sequence of the transcript with the known protein database [11]. LGC can characterize and identify lncRNAs based on the relationship between open read frame length and GC content, and can accurately distinguish lncRNAs from protein-coding RNA in a cross-species manner [12]. CNCI is a method to distinguish coding transcripts with non-coding ones by adjacent nucleotide triad features. The tool does not rely on known annotation files and can effectively predict incomplete transcripts and antisense transcripts. When the transcript score is < 0, it is noncoding [13]. CPAT is an analytical method to determine encoding and non-coding ability of transcripts by calculating Ficketter score and Hexmaer score based on ORF length and coverage [14]. In our analysis, a total of 10,343 known lncRNAs were detected and 2154 novel predicted lncRNAs were identified. Amongst them, a total of 546 lncRNAs were found to be dysregulated in CA specimens compared to adjacent mucosal tissues. In addition, PCA for all expressed mRNAs or lncRNAs and DEGs in CA separated from those in CON, indicating that HPV infection changed the expression pattern of host genes. Notably, the functional GO term and pathway analysis of DEGs coexpressed with DElncRNAs in CA were enriched in cell adhesion, keratinocyte differentiation, and the pathways involved in ECM-receptor interaction, PI3K/AKT signaling pathway, TGF-ß signaling pathway and local adhesion, which indicated that they played crucial roles in CA pathogenesis, consistent with our previous study [30].

For the lncRNA field, discerning functional noncoding RNAs from a vast transcriptome is a principal priority and challenge. It is a potential strategy to classify and elucidate lncRNA function based on the *cis*- or *trans*-regulatory pattern [18]. *Cis*-acting lncRNAs can be positioned and oriented relative to their nearby genes, such as lincRNAs around transcription factor start sites [31]. Furthermore, DEGs *cis*-regulated by these differential TFs (especially belonging to the HOX family) were enriched in GO terms of epithelial development and regulation of transcription or gene expression, which indicated the probable function of lncRNAs differentially expressed in CA.

We further confirmed the expression of 3 pairs of DElncRNAs and *cis*TFs, EVX1-AS and HOXA13, HOXA11-AS and EVX1, and DLX6-AS and DLX5. In our analysis, we chose the Pearson correlation coefficient > 0.6 and P value < 0.01 as set a cutoff to screen the lncRNA target pairs. Their PCR validation results were consistent with the sequencing data. For lncRNA EVX1-AS, there are very few data about its function. It was only reported that EVX1-AS might be correlated with colon cancer in the LncRNADisease database [32]. Although EVX1-AS and EVX1 have been reported to localize to the same genomic locus and are in fact transcribed from alternative promoters in close proximity [33, 34]. the Pearson correlation coefficient between EVX1 and EVX1-AS in our study was 0.59, and the P value was 0.071. Therefore, the pair of EVX1 and EVX1-AS was not included in our study. It is worth thinking about and confirm whether there is a better way to screen the pairs of lncRNA-cisTF and how to set the screen cutoff. Here, in our study, EVX1-AS and its *cis*-regulatory TF-HOXA13 both decreased in CA. HOXA13, a HOX transcription factor, usually functions as an oncogene [35]. In terms of HOXA13 in viral infection or inflammation, HOXA13 participates in inflammation activation. HBV DNA polymerase suppresses HBV replication via activation of the CREB1-HOTTIP-HOXA13 axis, followed by attenuation of HBV replication to result in persistent infection [36]. During influenza infection in the absence of the IFN-α/ß receptor, inflammatory and apoptotic responses may be initiated via induction of Ing1, Nr4a1, Polr2a, or Hoxa13 [37]. LncRNA-HOTTIP regulates the UV-mediated cellular response to UV through the coordinated activation of its neighboring gene Hoxa13 [38]. Thus, we speculated that attenuated EVX1-AS in CA might reduce the inflammatory response via *cis*-downregulated HOXA13 to allow HPV to evade host recognition. For lncRNA HOXA11-AS, a meta analysis showed up-regulated HOX11-AS in various cancers plays a tumor promoter [39], and indicates a risk factor for poor clinical outcome [40]. In the regulation of inflammation, HOXA11-AS mediated CaOx crystal-induced renal inflammation via the miR-124-3p/MCP-1 axis [41], while inhibition of HOXA11-AS repressed neuroinflammation and neuronal apoptosis in Parkinson’s disease model through through miR-124-3p-FSTL1-NF-κB axis [42] and miR-98-5p/EphA4 [43]. In addition, induction of HOXA11-AS by hypoxia/inflammation upregulated of HIF-1α and C/EBPβ further to promote epithelial-mesenchymal transition ability of nephroblastoma [44]. All these evidences indicate that HOXA-11 acts as a proinflammatory factor. In our results, whether the down-regulated HOX11-AS in CA also attenuates inflammation in CA to promote immune evasion, it needs further functional validation.

The expression of the pair of DLX6-AS and DLX5 increased in CA in the RNA-seq data and qRT‒PCR results. Both DLX6 and DLX-5 act as oncogenes to promote cellular proliferation and inhibit apoptosis [45–48]. Interestingly, DLX6-AS1 and DLX5 play their roles by targeting the PI3K/AKT/mTOR signaling pathway [49]. Whether increased DLX6-AS and DLX5 in CA participate in regulating the proliferation and apoptosis of HPV-infected cells needs to be confirmed by carrying out functional gain- and loss-of function experiments to explore the function of candidate lncRNAs in CA.

## Conclusions

CA has a specific lncRNA and mRNA profile, and dysregulated lncRNAs in CA may be potential novel candidates. Functional enrichment analysis revealed that the DEGs coexpressed with DElncRNAs were involved in cell adhesion, keratinocyte differentiation, and the pathways involved in ECM-receptor interaction, PI3K/AKT signaling pathway, TGF-ß signaling pathway and local adhesion. We further constructed a network among DElncRNAs-*cis*TFs-DEGs and found that some differential lncRNAs play *cis*-regulatory roles on TFs, especially those belonging to the HOX family. In the future, we will explore the relationships between the candidate lncRNAs of *EVX1-AS, HOXA11-AS, DLX6-AS* and CA and their probable regulatory mechanisms. It will be useful to understand the pathogenesis of CA to provide new directions for the prevention, clinical treatment and efficacy evaluation of CA.

### Electronic supplementary material

Below is the link to the electronic supplementary material.

Supplementary Material 1

## Data Availability

Sequence data that support the findings of this study have been deposited in GEO database with the accession number GSE172140.

## Abbreviations

CA: Condyloma acuminatum
CNCI: Coding-Non-Coding Index
CPC2: Coding Potential Calculators
CPAT: Coding Potential Assessment Tool
DEG: Differentially expressed gene
GO: Gene Ontology
KEGG: Kyoto Encyclopedia of Genes and Genomes
LGCl: ORF Length and GC Content
lncRNA: Long noncoding RNAs
PCA: Principal component analysis
RPM: Reads per million mapped reads
RT-qPCR: Real-time quantitative reverse transcriptase polymerase chain reaction
TF: Transcription factor
