# Supplementary material for: Expression profile of long noncoding RNAs and comprehensive analysis of lncRNA-cisTF-DGE regulation in condyloma acuminatum
Source: BMC Med Genomics. 2024 Jun 20;17:167. doi: 10.1186/s12920-024-01938-z (PMC11188504; doi:10.1186/s12920-024-01938-z)
Supplement: Supplementary file 1 — Supplementary Material 1 [file 12920_2024_1938_MOESM1_ESM.docx]

Supplementary material


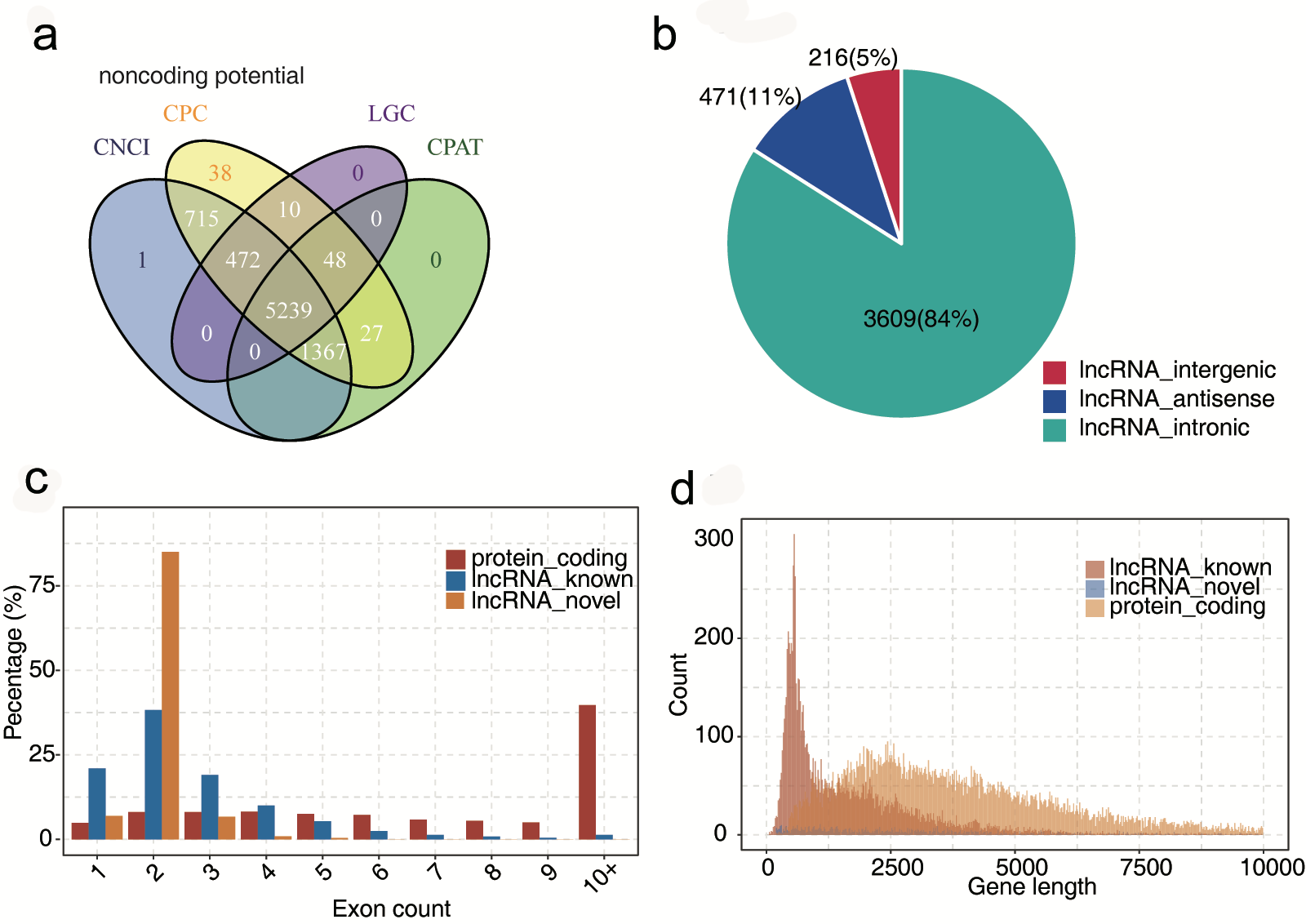


Fig. S1 Analysis of lncRNA and mRNA expression profile in condyloma acuminatum. (**a**) Venn diagram showing the overlapping lncRNAs by four methods. (**b**) Pie chart showing the lncRNA type distribution. (**c**) Distribution of exon lengths of known lncRNAs, novel lncRNAs, and protein-coding RNAs. (**d**) Density of the length distribution of known lncRNAs, novel lncRNAs, and protein-coding RNAs.


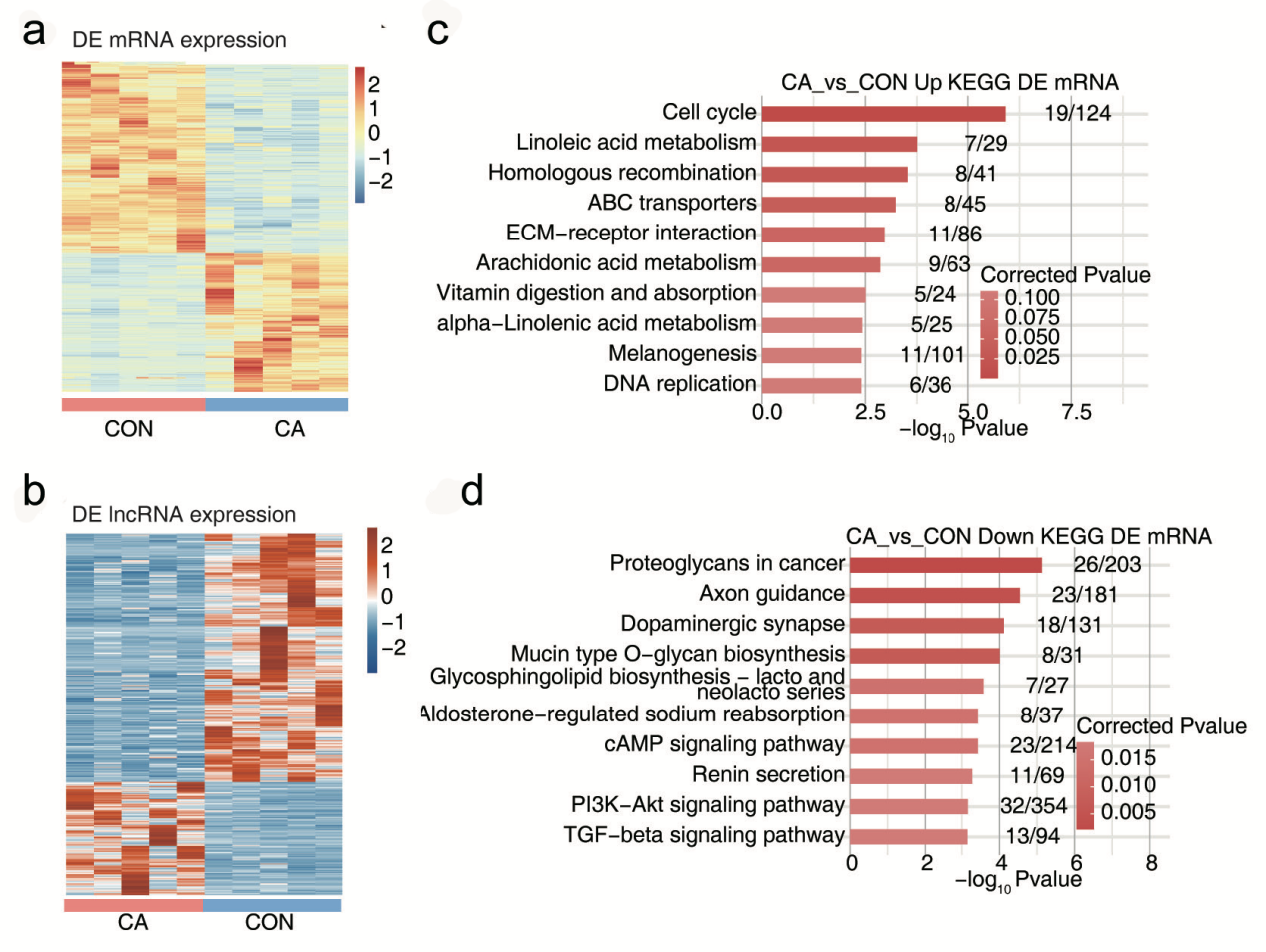


Fig. S2 Analysis of differential expression of lncRNAs in condyloma acuminatum. (**a-b**) Hierarchical clustering heatmap showing the expression levels of DEmRNAs and DElncRNAs. (c-d) Bar plot showing the top 10 enriched KEGG pathways of the upregulated and downregulated DEGs.


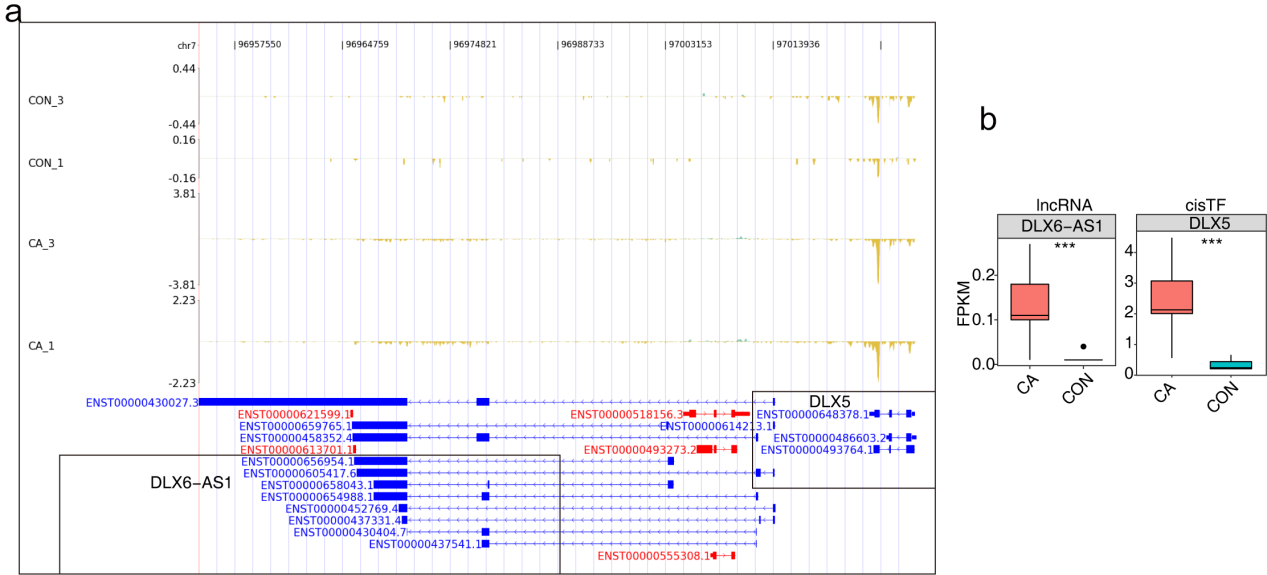


Fig. S3 Exhibition of the differential expression of lncRNAs, *cis*TFs and targeted DEGs in CA and CON samples. (**a**) Read distribution showing lncRNA DLX6-AS1 and its *cis*-regulated TF DLX5. The upward and green reads in the upper part represent for the red transcripts in the bottom part, and the downward and yellow reads represent for the blue transcripts in the bottom part. (**b**) Boxplot showing the relative expression of lncRNA DLX6-AS1 and *cis*TF DLX5. *** *P* value < 0.001.
